# Establishing and Validating a Morphological Prediction Model Based on CTA to Evaluate the Incidence of Type-B Dissection

**DOI:** 10.3390/diagnostics13193130

**Published:** 2023-10-05

**Authors:** Yan Fu, Siyi Huang, Deyin Zhao, Peng Qiu, Jiateng Hu, Xiaobing Liu, Xinwu Lu, Lvfan Feng, Min Hu, Yong Cheng

**Affiliations:** 1 Department of Nursing, Shanghai Ninth People’s Hospital, Shanghai JiaoTong University School of Medicine, Shanghai 200011, China; leandra77@163.com (Y.F.);; 2 Second Ward of General Surgery, Suzhou Hospital of Anhui Medical University (Suzhou Municipal Hospital of Anhui Province), Suzhou 234000, China; crazyrobot@sohu.com; 3 Department of Vascular Surgery, Shanghai Ninth People’s Hospital, Shanghai Jiao Tong University School of Medicine, Shanghai 200011, China; 4 Shanghai Health Development Research Center (Shanghai Medical Information Center), Shanghai 200031, China

**Keywords:** type-B dissection, morphological prediction model, CTA, total thoracic aorta, aortic arch

## Abstract

**Background:** Many patients with Type B aortic dissection (TBAD) may not show noticeable symptoms until they become intervention and help prevent critically ill, which can result in fatal outcomes. Thus, it is crucial to screen people at high risk of TBAD and initiate the necessary preventive and therapeutic measures before irreversible harm occurs. By developing a prediction model for aortic arch morphology, it is possible to accurately identify those at high risk and take prompt action to prevent the adverse consequences of TBAD. This approach can facilitate timely the development of serious illnesses. **Method:** The predictive model was established in a primary population consisting of 173 patients diagnosed with acute Stanford TBAD, with data collected from January 2017 and December 2018, as well as 534 patients with healthy aortas, with data collected from April 2018 and December 2018. Explicitly, the data were randomly separated into the derivation set and validation set in a 7:3 ratio. Geometric and anatomical features were extracted from a three-dimensional multiplanar reconstruction of the aortic arch. The LASSO regression model was utilized to minimize the data dimension and choose relevant features. Multivariable logistic regression analysis and backward stepwise selection were employed for predictive model generation, combining demographic and clinical features as well as geometric and anatomical features. The predictive model’s performance was evaluated by examining its calibration, discrimination, and clinical benefit. Finally, we also conducted internal verification. **Results:** After applying LASSO logistic regression and backward stepwise selection, 12 features were entered into the prediction model. Age, aortic arch angle, total thoracic aorta distance, ascending aorta tortuosity, aortic arch tortuosity, distal descending aorta tortuosity, and type III arch were protective factors, while male sex, hypertension, aortic arch height, and aortic arch distance were risk factors. The model exhibited satisfactory discrimination (AUC, 0.917 [95% CI, 0.890–0.945]) and good calibration in the derivation set. Applying the predictive model to the validation set also provided satisfactory discrimination (AUC, 0.909 [95% CI, 0.864–0.953]) and good calibration. The TBAD nomogram for clinical use was established. **Conclusions:** This study demonstrates that a multivariable logistic regression model can be used to predict TBAD patients.

## 1. Introduction

Acute Stanford type B dissection is a prevalent condition, accounting for 25–40% of all cases of thoracic aortic dissection (TBAD) [1]. Great progress has been made in the past 20 years in surgical treatment, graft design, and imaging technology [2]; it is still a vascular disease seriously endangering life and health, and the in-hospital mortality of TBAD patients remains at 9.3% [3]. Before becoming ill, many patients with TBAD do not exhibit any noticeable clinical symptoms. As a result, when they do become sick, they often present with severe and life-threatening symptoms [4]. Hence, the main goal for TBAD prevention and treatment is to identify the high-risk groups of patients prone to TBAD before irreversible damage occurs [5].

With advancements in CT technology, especially the development of three-dimensional reconstruction techniques for aortic CTA, our understanding of the morphology of the aorta is gradually deepening. Previous studies have shown that aortic length has better predictive efficacy compared to diameter. Furthermore, by incorporating both dimensions of morphological indices, the Aortic Health Index (AHI) has been developed as a tool to predict Type A aortic dissection. This morphological index has been made clinically available as a predictive tool [6]. However, the dimensions of these length predictions for the aortic layers are still limited. Existing morphological parameters of the aorta either rely on overly simplistic imaging measurements, lacking a comprehensive quantification of the overall aortic morphology, or utilize overly cumbersome analysis of imaging information, neglecting clinical practicality and interpretability. 

It is possible to identify patients at risk for TBAD by analyzing morphological risk factors, as the degradation of aortic morphology can cause abnormal biomechanical changes that contribute to the development of TBAD [7].

The previous guidelines for aortic dissection suggested that when, among morphological risk factors, the transverse diameter of the ascending aorta exceeds 5.5 cm, patients should receive preventive treatment to prevent TBAD [8]. Nevertheless, ample evidence suggests that the increase in aortic diameter is not strongly correlated with TBAD development. Researchers reexamined the medical records of patients with type A aortic dissection from the International Registry of Acute Aortic Dissection (IRAD) and found that among these patients, 60% had an aortic diameter less than 5.5 cm and 40% had a diameter less than 5 cm. Observing the diameter of the aorta can result in the misdiagnosis of a majority of high-risk individuals with aortic dissection [9]. Recently, a study has put forward aortic length as an alternative measure of aortic expansion, which could potentially serve as a predictor for aortic rupture or dissection [10].

Various risk factors, including hypertension, aging, atherosclerosis, and other diseases, are prevalent among a large segment of the population, contributing to an increased likelihood of developing TBAD [11]. We cannot protect all these people in an effort to prevent TBAD, which is a disease with a low incidence rate. We expect that by utilizing the morphological prediction model of the aortic arch, it would be possible to accurately identify and protect individuals at high risk before the onset of symptoms [12]. Due to the scarcity of imaging data before the onset of TBAD, we collected data for a cross-sectional study from TBAD patients receiving thin-section (0.6 mm) angiography or enhanced CT and healthy patients.

Our study used a morphological index of the aortic arch as a predictor of TBAD. Then, we observed that the relative length has a different prediction ability for TBAD in the recognition group.

## 2. Methods

### 2.1. Patient Sampling

We followed a retrospective, cross-sectional research design.

TBAD patients received an acute Stanford TBAD diagnosis at one institution in China. We retrospectively analyzed TBAD patients who received contrast-enhanced CT angiography with thin-cut (0.6 mm) images. This study included patients treated from January 2017 until December 2018.

The exclusion criteria for the TBAD group were as follows: (1) connective tissue disease (Marfan’s, Loeys–Dietz, or Ehlers–Danlos syndrome); (2) traumatic dissections or inflammatory dissections; (3) other aortic diseases, including aneurysms; (4) history of aortic surgery; (5) previous cardiothoracic disease or surgery; (6) diseases that may distort the thoracic aortic anatomy (pulmonary nodule diameter > 3 cm, mediastinal masses or lymph node diameter > 1 cm, pneumothorax, pulmonary bullae diameter > 3 cm, previous thoracic and mediastinal surgery, etc.); and (7) diseases that may alter the thoracic wall structure (scoliosis, barrel chest, pectus carinatum, previous spinal surgery, etc.).

We retrospectively evaluated consecutive healthy aorta patients who underwent thin-cut (0.6 mm) contrast-enhanced CT angiography or contrast-enhanced chest CT in the same institution between April 2018 and December 2018 for inclusion in the healthy control group. The exclusion criteria for the healthy control group were as follows: (1) suspected or diagnosed aortic disease; (2) history of cardiothoracic disease or surgery; (3) diseases that may distort thoracic aortic anatomy; and (4) diseases that may distort the thoracic wall structure.

### 2.2. Outcomes

We collected demographic and clinical characteristics from the electronic records of outpatients (the healthy control group) and inpatients (the TBAD group). The methods used complied with the STROBE guidelines and the Declaration of Helsinki and gained ethical approval from the participating institution (SH9H-2019-T144-2). Written consent from patients was not required due to the retrospective design of this investigation. This work is registered with the Chinese Clinical Trial Registry under ChiCTR2000029219.

### 2.3. Predictor Variables

The shape of the aorta is composed of various dimensions. In a previous study, we divided the aorta into four segments [10]. We measured each segment, including its length, twist, and other indicators, to describe the local shape of the individual aortic segments. We think this is crucial for analyzing aortic dissection etiology, especially in the aortic arch. For the aortic arch, we measured a series of indicators, such as the angle of its arch, width, and height. Furthermore, we employed metrics such as the overall length and distortion of the aorta to characterize its overall shape.

Relevant and anonymous Standard Digital Imaging and Communications in Medicine (DICOM) data were retrieved for analysis. Three-dimensional multiplanar reconstruction was carried out via EndoSize software version 3.1 (Rennes, France). A generated centerline connected the sinotubular junction to the descending thoracic aorta diaphragmatic location. In TBAD data, the observer manually placed centerline seed points at the artery lumen (including both true and false lumens) center. According to a prior investigation [13], there can be four sections to the thoracic aorta, 90° to the centerline. These segments included the ascending aorta, the aortic arch, the proximal descending thoracic aorta, and the distal descending thoracic aorta.

**Figure 1:**
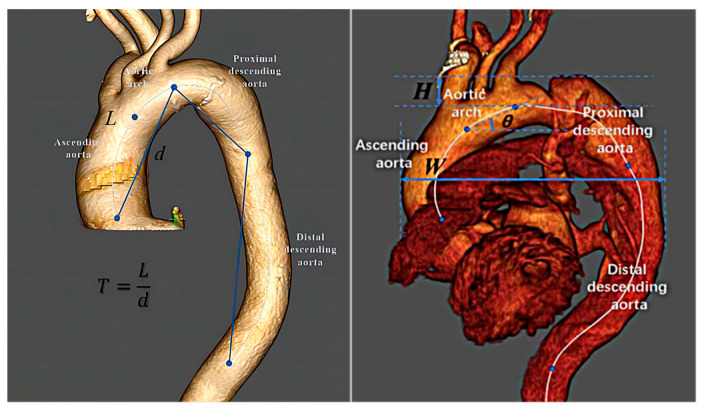
Thoracic aorta depicting study measurements. The thoracic aorta was separated into four portions: the ascending aorta, the aortic arch, the proximal descending thoracic aorta, and the distal descending thoracic aorta. Tortuosity (T) represents the ratio of the line path length (L) center to the direct linear distance between its two endpoints (d). Arch width (W) refers to the maximal distance between the outer curvature of the ascending aorta and the descending aorta. Arch height (H) represented the vertical distance between the brachiocephalic artery origin and arch vertex. The arch angle (θ) represented the angle between the line connecting the brachiocephalic and left subclavian artery origins and the horizontal line.

The tortuosity (T) was calculated as the ratio of the center line path length (L) to the direct linear distance between its two endpoints (d). The arch width (W) was described as the maximal distance between the outer curvature of the ascending aorta and the descending aorta. The arch height (H) refers to the vertical distance between the brachiocephalic artery origin and arch vertex. The arch angle (θ) represented the angle between the line connecting the brachiocephalic and the left subclavian artery origins and the horizontal line. The individual segment length was computed as the distance along the centerline between the aforementioned points. (Figure 1)

Aortic arch parameter measurements were taken from the aortic perspective. The arch width was described as the maximal distance between the outer curvature of the ascending aorta and the descending aorta. The arch height represented the vertical distance between the brachiocephalic artery origin and the arch peak. The arch angle represented the angle between the line connecting the brachiocephalic and left subclavian artery origins and the horizontal line. We employed Heuts’ retrospective modeling system to achieve normalized pre-evaluation dimensions. According to Rylski et al., the descending aorta length increases by 3% following dissection; however, the ascending aorta length and aortic arch remain the same. Herein, we establish a model of the aorta of TBAD patients as a preanatomic length by subtracting 3% of the descending aorta length.

### 2.4. Statistical Description

Continuous and categorical data are provided as the mean ± standard deviation (SD) and numbers + percentages, respectively. Data normality was assessed via the Shapiro—Wilk test, and data homogeneity was assessed using Levene’s test. Student’s *t* test, Welch’s *t* test, and the Mann-Whitney test were used to compare continuous data between TBAD and control participants as well as between the derivation set and validation set. The Chi-square test was used to compare categorical data between the two cohorts. The primary data does not contain any missing values.

### 2.5. Derivation of the Model

In the pre-analysis, all variables were included in the multivariable logistic regression analysis, and the VIF (variance inflation factor) test was used, indicating collinearity issues. Therefore, LASSO regression is used to solve collinearity problems and select the most valuable predictive profiles from the derivation cohort [14]. Subsequently, using multivariable logistic regression analysis, we established a predictive model to estimate TBAD risk. Backward stepwise selection was implemented by employing the likelihood ratio test following Akaike’s guidelines as the stopping criterion [15]. Risks were stated as odds ratios (ORs) accompanied by 95% confidence intervals (CIs). 

To give clinicians a quantifiable means of predicting the likelihood of TBAD in individuals, we constructed the TBAD nomogram using multivariable analysis of the developmental cohort.

### 2.6. Prediction Model Performance in the Derivation Set

Calibration curves, along with the Hosmer–Lemeshow test, were used to evaluate the prediction model calibration. (Significance indicated that the model was not perfectly calibrated) [16]. To quantify the discrimination performance of the prediction model, the receiver operating characteristic (ROC) curve was used.

### 2.7. Internal Validation of the Model

Prediction model internal validation utilized the validation set. The derivation set logistic regression formula was applied to the validation set, and the TBAD probability was computed for individual patients. Then, the ROC curve and calibration curve for the validation set were obtained.

The Delong test was used to compare ROC curve differences between the derivation set and validation set. (Significance indicated that two ROC curves were different.) 

Statistical analysis was performed with R software (version 4.2.1). All *p* values tested were two-sided. *p* < 0.05 was set as the significance threshold.

## 3. Results

### 3.1. Baseline Profiles

In all, we recruited 173 TBAD patients and 534 healthy controls. Patients in the TBAD group were typically younger in age (60.23 ± 12.33 vs. 65.24 ± 12.21 years, *p* < 0.001). The number of men in the TBAD group was higher than that in the healthy control group. Patients in the TBAD group had a higher average BMI than those in the other groups. (25.22 ± 3.55 vs. 23.52 ± 3.83 kg/m^2^, *p* < 0.001). Patients in the TBAD group had a higher BSA (1.73 ± 0.14 vs. 1.66 ± 0.16 m^2^, *p* < 0.001). The TBAD group had patients with higher hypertension (77.46% vs. 37.08%, *p* < 0.001), more patients with diabetes mellitus diagnoses (21.39% vs. 15.73%, *p* = 0.109), and patients who displayed stronger smoking habits (35.26% vs. 31.65%, *p* = 0.431) relative to the healthy control group. Table 1 summarizes the baseline profiles of TBAD patients and the healthy control group of patients.

The derivation set and validation set included 496 and 211 patients, respectively. The average ages in the derivation set and validation set were 63.86 and 64.36 years, respectively. TBAD baseline data are available in Table 2. Moreover, no marked difference was detected in the baseline characteristics between the aforementioned sets.

### 3.2. Aortic Geometry

There were significant differences in most variables between TBAD patients and a healthy control group, except for some indicators, such as ascending aorta, total thoracic aorta, distal descending aorta, and type III arch, which are shown in Table 3. No marked difference was detected in the baseline characteristics between the derivation set and validation set (Table 4).

### 3.3. Feature Selection

**Figure 2:**
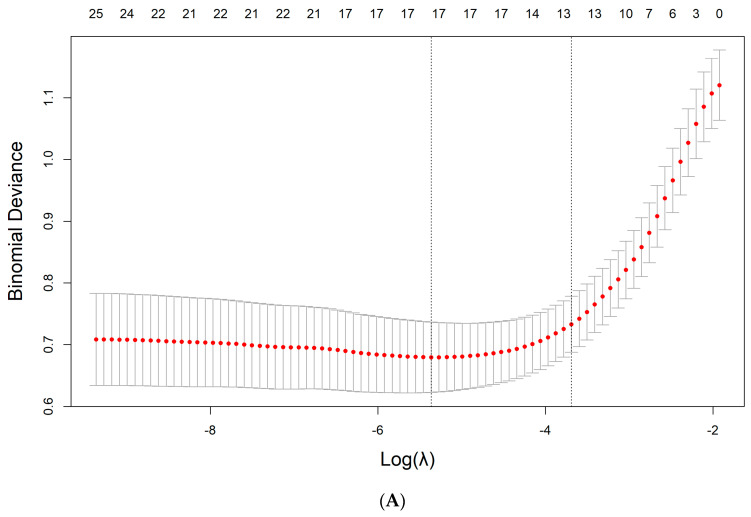

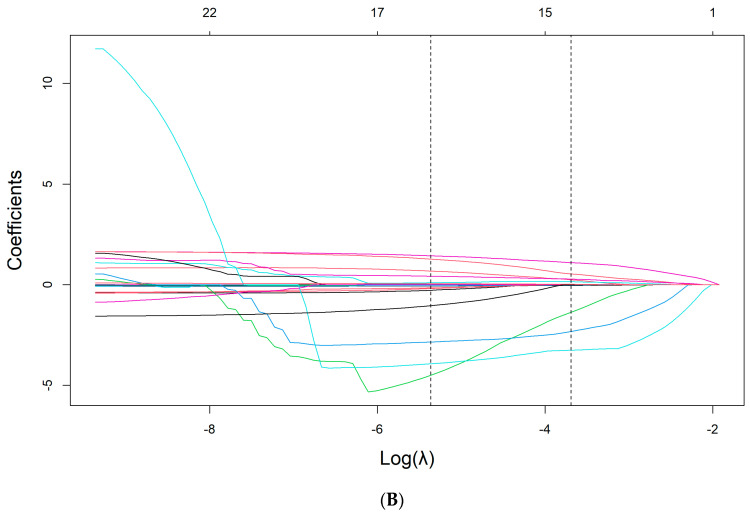
Feature selection via the least absolute shrinkage and selection operator (LASSO) binary logistic regression model. (**A**) The binomial deviance was generated against log (λ). We employed minimal criteria and 1 standard error (the 1−SE criteria) to generate dotted vertical lines at optimal values. Using the 0.005 λ value for log (λ), we chose −5.364 (minimal criteria). (**B**) LASSO characteristics of 27 profiles. A coefficient profile plot was generated versus the log (λ) sequence. We employed optimal λ, providing 17 nonzero coefficients, to draw a vertical line at the selected value.

A total of 27 features were reduced to 17 potential predictors on the basis of 496 patients in the derivation set (Figure 2A,B) and were features with nonzero coefficients in the LASSO logistic regression model.

### 3.4. Prediction Model for TBAD

After backward stepwise selection, 12 features were entered into the prediction model (Table 5). We could discover through VIF that there is no collinearity problem. Meanwhile, we found that age, aortic arch angle, distance of the total thoracic aorta, AA tortuosity, aortic arch tortuosity, distal DA tortuosity, and type III arch were protective factors, while male sex, hypertension, aortic arch height, and distance of the aortic arch were risk factors.

### 3.5. Performance of the Prediction Model for TBAD

**Figure 3:**
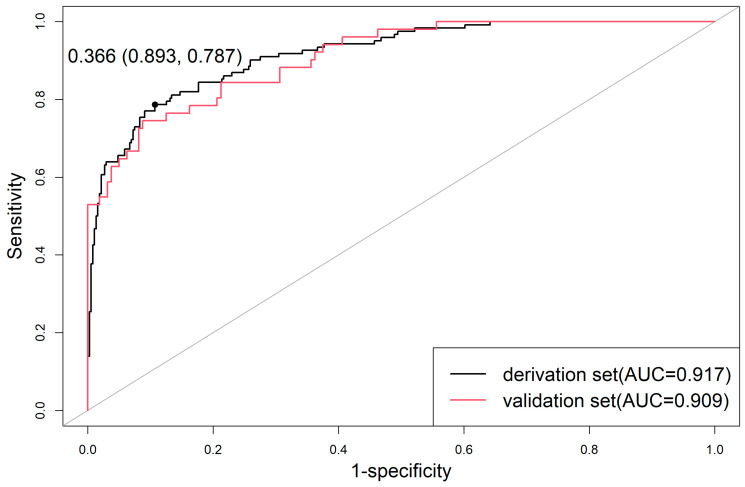
The ROC curve is constructed by the derivation and validation sets.

The probability of TBAD in each patient was calculated by a multivariable logistic regression model, and the ROC curve was constructed by combining the actual occurrence of TBAD. It could be seen from the ROC curve constructed by the derivation set that the optimal probability critical point for judging whether a TBAD event occurred was 0.366, and the AUC reached 0.917 (95% CI, 0.890–0.945) (Figure 3).

The prediction model calibration curve for TBAD probability exhibited satisfactory agreement between estimated and actual values in the derivation set. The Hosmer–Lemeshow test demonstrated no significance (*p* = 0.488), which suggested that there was no departure from a perfect fit.

### 3.6. Evaluation of Model Prediction Ability

**Figure 4:**
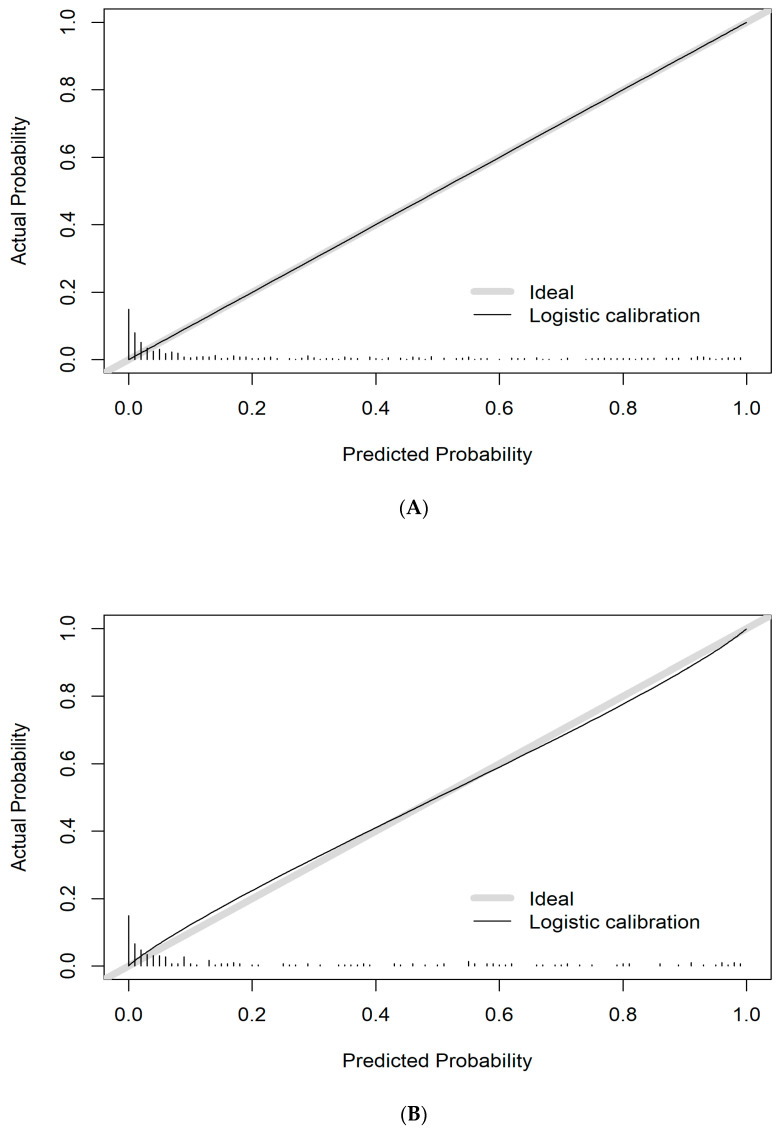
The prediction model has calibration curves. (**A**) The derivation cohort prediction model calibration curve. (**B**) The validation cohort prediction model calibration curve. Calibration curves demonstrate the agreement between the estimated and actual TBAD outcomes. The y-axis depicts the observed TBAD possibility. The x-axis depicts the estimated TBAD possibility. The diagonal gray line shows a perfect estimation as suggested by an ideal model. The black solid line exhibits the prediction model performance, and a close fit to the diagonal gray line indicates augmented estimation.

The validation set exhibited satisfactory calibration for TBAD probability (Figure 4B). The Hosmer–Lemeshow test showed no significance (*p* = 0.173). Additionally, the prediction model AUC for estimating TBAD in the validation set was 0.909 (95% CI, 0.864–0.953) (Figure 3). The Delong test showed no significance (*p* = 0.748). 

### 3.7. Clinical Use

The TBAD nomogram was developed in the derivation set. Clinicians can use the TBAD nomogram to predict the individual probability of TBAD.

## 4. Discussion

Aortic dissection is asymptomatic before onset; therefore, it is meaningful to screen the population before onset, but how to screen is a challenging thing. First, the incidence rate of aortic dissection is very low. When screening a large population, it is necessary to have a large sample size to identify and eliminate high-risk factors. Only 155 patients were discovered in the Oxford Vascular Research study conducted over 10 years [17]. Therefore, traditional epidemiology can only screen out common cardiovascular risk factors, and it is difficult to screen high-risk factors for aortic dissection. In contrast, there are two factors that contribute to the occurrence of TBAD. The initial factor is the occurrence of pathological shape changes in the vascular media before TBAD onset [18]. Aortic wall structural weakness and enhanced wall tension Several connective tissue components contribute to TBAD etiology, and multiple connective tissue diseases, such as Marfan and Ehler-Danlos syndromes, are critical predisposing agents. Second, the beginning of TBAD is linked to the strength exerted by the blood flow (including absolute blood pressure, pulse pressure, and dP/dT) [19]. Hence, it is possible to identify the risk factors for TBAD based on aortic geometry. Earlier research indicated that the risk factors could be associated with the size of the aorta [20,21,22].

However, the results of many studies have refuted this view, indicating that most acute type B aortic dissection patients exhibit a descending aortic diameter < 5.5 cm prior to dissection and are therefore not within the criteria for elective descending thoracic aorta repair [23,24]. Aortic diameter measurements are not efficient in approaches to preventing aortic dissection; thus, TBAD will be missed in many patients, and other methodologies are warranted to screen patients at enhanced risk for acute TBAD. In a prior investigation, we found that the distorted shape of the proximal descending aorta and the aortic arch shape of TBAD had a certain predictive effect on the incidence of dissection. However, in our TBAD data, we found that aortic length could not predict TBAD alone; therefore, we need to identify morphological risk factors apart from aortic diameter and length to predict TBAD.

In the current retrospective study, we applied the LASSO logistic regression model to demographic information and geometric and anatomical variables. We sought to predict dissection using variables screened by the LASSO logistic regression model. A nomogram was created to provide a scoring system for assessing aortic dissection. The resulting prediction model incorporated 12 features. We found that age, aortic arch angle, total thoracic aorta distance, ascending aorta tortuosity, aortic arch tortuosity, distal descending aorta tortuosity, and type III arch were protective factors, while male sex, hypertension, aortic arch height, and aortic arch distance were risk factors. 

Mehta et al. used a logistic regression model to assess in-hospital death among type A dissection patients who were recruited in the International Registry of Acute Aortic Dissection. Their findings revealed that age ≥ 70 years, sudden onset of chest pain, hypotension, kidney failure, pulse deficit, and abnormal electrocardiogram (ECG) results were predictive factors for death during hospitalization [25,26]. The relatively low AUC value (0.74) indicates that the robustness of this model is limited. Using the same database of TBAD, Tolenaar and colleagues analyzed the clinical features of 1034 patients and developed a bedside risk prediction tool for in-hospital mortality [27]. Multiple machine learning algorithms were applied in a single-center retrospective cohort study to forecast the mortality of acute TBAD patients in hospitals. This study found that the most significant factors affecting mortality were treatment methods, the type of acute aortic dissection, and levels of ischemia-modified albumin [28]. Because treatment type is one of the main factors in the prediction model, this conclusion may not be applicable to TBAD patients who recently received hospital admission but have not received any treatment. The multivariable logistic regression model included demographic information and geometric and anatomical variables and exhibited better prediction efficiency than previously reported. 

**Figure 5:**
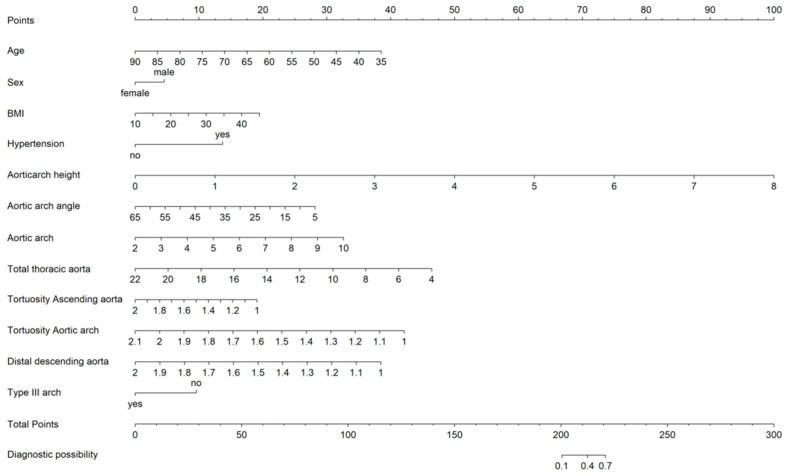
The TBAD nomogram.

For this, the AUC reached 0.917 (95% CI, 0.890–0.945), indicating that the integration of parameters highly benefited TBAD prognosis prediction efficacy. We can utilize logistic regression to calculate the TBAD nomogram (Figure 5) to determine the risk score as a supplementary approach to hospital treatment. In addition, calibration curves were generated to represent participant estimates. As we can see, the calibration intercept of the multivariable logistic regression model equals 0, suggesting robust multivariable model performance at the boundary point. Our findings suggest that the multivariable logistic regression model performed with great accuracy and robustness in predicting the occurrence of acute TBAD. Additionally, we developed a nomogram based on this model, which can serve as a complementary prediction method. 

During our analysis, we found that the overall shape and local area of the aorta would produce too many feature selections. Therefore, we needed to select meaningful TBAD predictions in features. In our research, 27 features were reduced to 12 potential predictors in the derivation set, and we tried to predict the occurrence of TBAD and achieved good results. Hypertension is associated with aortic wall stress and is a risk factor for TBAD. Participants who died of TBAD were younger and more likely to be males than those without TBAD.

Additionally, TBAD is correlated with aortic arc elongation. The aforementioned evidence indicates that the aortic arch morphology and nearby aortic segments may be an aortic dissection sign. Nevertheless, prior studies were focused primarily on investigating the aortic diameter and length, and the association between TBAD and aortic arch geometry (such as the width, height, and angle) was not extensively examined. 

This and other studies confirm that aortic dissection is related to aortic morphological changes. The findings from this research can offer guidance for further investigation in the future. In the following studies, we hope to find the best predictor of TBAD incidence, which we expect to be a simple parameter describing the aortic arch shape. High-risk groups of TBAD, we may prevent patients from developing TBAD in the early stages because the time of morphological deterioration will not cause TBAD. In the case of patients with aberrant aortic arch morphology, outpatient visits need to be increased. Antihypertensive drugs must be used to rigorously manage blood pressure and eliminate any other potential risk factors for these particular patients. Therefore, additional investigations are warranted to validate our results. In upcoming experiments, we recommend identifying hemodynamic and histological risk indicators with numerical and experimental evidence. 

Herein, the healthy patient group consists of individuals who were assessed in the radiology department for unrelated medical conditions rather than being chosen from a group of volunteers specifically deemed healthy.

Based on the conclusions of our article, we have the potential to bring all patients who have undergone chest CT scans, such as those with lung diseases, to be screened for TBAD and diagnosed using the model. When we identify risk factors in a patient, the next step is to conduct a cost-effectiveness assessment based on health economics to calculate the intervention costs for different patients and evaluate the benefits. For example, if the risk of TBAD exceeds a certain threshold, we would implement corresponding measures such as prescribing antihypertensive medication, closely monitoring the patient, and even considering surgical intervention. However, several limitations need to be considered. The cohort of healthy aortic artery patients selected in this study was derived from a cohort formed by the radiology department for the evaluation of other diseases, rather than being selected from a group of healthy volunteers. Although this study excluded aortic diseases, heart diseases, and other conditions that could potentially affect aortic morphology, there may still be other biases present in this study. A prospective lifelong cohort study on a group of healthy volunteers could provide more convincing evidence. 

Our team is parsing the data of 50 thousand healthy people in the biobank to find the high-risk factors for TBAD, including exposure factors such as genes and living habits. However, Biobank lacks imaging data of the aorta, and in our next research project, we hope to collect imaging data from healthy people and establish a more accurate dissection prediction scheme. 

## 5. Conclusions

In conclusion, this study constructed a multivariable logistic regression model for predicting TBAD patients. The model exhibited great prediction accuracy. The results demonstrated that features such as: Ascending aorta, Aortic arch, Proximal descending aorta, Distal descending aorta, Total thoracic aorta, Aortic arch height, Aortic arch width, Aortic arch angle, Ascending aorta, Aortic arch, Distal descending aorta, Total thoracic aorta, Ascending aorta, Aortic arch, Distal descending aorta, Total thoracic aorta, Type III arch. These models need to be validated in a prospective, large-sample database.

## Figures and Tables

**Table 1:** Baseline demographics of TBAD patients and the healthy control group.

	Studies *n* = 173	Controls *n* = 534	*p*
Male (*n*, %)	142 (82.08)	312 (58.43)	<0.001
Age (y)	60.23 ± 12.33	65.24 ± 12.21	<0.001
BMI (kg/m^2^)	25.22 ± 3.55	23.52 ± 3.83	<0.001
BSA (m^2^)	1.73 ± 0.14	1.66 ± 0.16	<0.001
Hypertension (*n*, %)	134 (77.46)	198 (37.08)	<0.001
Diabetes (*n*, %)	37 (21.39)	84 (15.73)	0.109
Smoking (*n*, %)	61 (35.26)	169 (31.65)	0.431

Values are expressed as the mean ± SD. BMI Body mass index BSA Body surface area.

**Table 2:** Baseline demographics of the derivation and validation sets.

	All *n* = 707	Derivation Set *n* = 496	Validation Set *n* = 211	*p*
Male (*n*, %)	454 (64.21)	323 (65.12)	131 (62.09)	0.494
Age (y)	64.01 ± 12.42	63.86 ± 12.43	64.36 ± 12.42	0.628
BMI (kg/m^2^)	23.94 ± 3.83	24.01 ± 3.85	23.77 ± 3.79	0.460
BSA (m^2^)	1.68 ± 0.16	1.68 ± 0.16	1.66 ± 0.16	0.145
Hypertension (*n*, %)	332 (46.96)	234 (47.18)	98 (46.45)	0.924
Diabetes (*n*, %)	121 (17.11)	88 (17.74)	33 (15.64)	0.569
Smoking (*n*, %)	230 (32.53)	161 (32.46)	69 (32.7)	1.000

Values are provided as the mean ± SD. BMI Body mass index BSA Body surface area.

**Table 3:** Geometric and anatomical variables among TBAD patients and healthy controls.

	Studies *n* = 173	Controls *n* = 534	*p*
Length			
Ascending aorta	62.49 ± 11.25	61.8 ± 11.09	0.480
Aortic arch	27.68 ± 6.81	25.54 ± 7.47	0.001
Proximal descending aorta	74.92 ± 16.83	68.14 ± 15.67	<0.001
Distal descending aorta	140.6 ± 19.81	152.05 ± 23.44	<0.001
Total thoracic aorta	305.69 ± 33.63	307.53 ± 38.04	0.437
Aortic arch height, mm	2.49 ± 1.04	1.84 ± 0.89	<0.001
Aortic arch width, mm	8.09 ± 1.63	7.2 ± 1.40	<0.001
Aortic arch angle	31.78 ± 10.78	33.62 ± 9.40	0.045
Distance			
Ascending aorta	7.24 ± 1.17	6.70 ± 0.95	<0.001
Aortic arch	6.16 ± 1.29	5.27 ± 1.07	<0.001
Distal descending aorta	12.23 ± 1.49	12.27 ± 1.59	0.801
Total thoracic aorta	11.40 ± 1.49	11.63 ± 1.78	0.240
Tortuosity			
Ascending aorta	1.25 ± 0.10	1.31 ± 0.13	<0.001
Aortic arch	1.22 ± 0.12	1.29 ± 0.15	<0.001
Distal descending aorta	1.15 ± 0.10	1.24 ± 0.14	<0.001
Total thoracic aorta	2.72 ± 0.45	2.69 ± 0.48	0.442
Type III arch (*n*, %)	38 (21.97)	98 (18.35)	0.349

Values are provided as the mean ± SD.

**Table 4:** Geometric and anatomical variables are among the derivation and validation sets.

	All *n* = 707	Derivation Set *n* = 496	Validation Set *n* = 211	*p*
Length				
Ascending aorta	61.97 ± 11.12	62.2 ± 11.25	61.41 ± 10.82	0.978
Aortic arch	26.06 ± 7.37	25.88 ± 7.59	26.50 ± 6.82	0.282
Proximal descending aorta	69.80 ± 16.21	69.73 ± 16.11	69.96 ± 16.48	0.868
Distal descending aorta	149.25 ± 23.12	149.75 ± 23.43	148.07 ± 22.38	0.369
Total thoracic aorta	307.08 ± 36.99	307.57 ± 37.34	305.94 ± 36.21	0.590
Aortic arch height, mm	2.00 ± 0.97	2.02 ± 1.00	1.97 ± 0.89	0.526
Aortic arch width, mm	7.42 ± 1.51	7.44 ± 1.51	7.37 ± 1.51	0.585
Aortic arch angle	33.17 ± 9.78	32.97 ± 9.91	33.63 ± 9.48	0.406
Distance				
Ascending aorta	6.83 ± 1.03	6.83 ± 1.06	6.83 ± 0.97	0.911
Aortic arch	5.49 ± 1.19	5.49 ± 1.19	5.47 ± 1.20	0.833
Distal descending aorta	12.26 ± 1.57	12.32 ± 1.60	12.11 ± 1.50	0.096
Total thoracic aorta	11.58 ± 1.71	11.67 ± 1.74	11.35 ± 1.63	0.054
Tortuosity				
Ascending aorta	1.29 ± 0.13	1.29 ± 0.14	1.29 ± 0.11	0.790
Aortic arch	1.28 ± 0.15	1.27 ± 0.15	1.28 ± 0.15	0572
Distal descending aorta	1.22 ± 0.14	1.22 ± 0.14	1.23 ± 0.13	0.493
Total thoracic aorta	2.7 ± 0.47	2.68 ± 0.46	2.75 ± 0.51	0.108
Type III arch	136 (19.24)	96 (19.35)	40 (18.96)	

Values are provided as the mean ± SD.

**Table 5:** Multivariable logistic regression analysis.

Variables	Estimate	Std. Error	Z Value	*p* Value	OR	95% CI		VIF
						Lower	Upper	
Age	−0.075	0.015	−4.857	0.000	0.928	0.899	0.956	1.75
Sex (Male)	0.839	0.364	2.307	0.021	2.314	1.147	4.797	1.17
BMI	0.070	0.044	1.604	0.109	1.072	0.985	1.168	1.11
Hypertension	1.533	0.325	4.712	0.000	4.631	2.483	8.929	1.24
Aortic arch height, mm	1.698	0.283	6.005	0.000	5.461	3.213	9.760	3.56
Aortic arch angle	−0.070	0.023	−3.005	0.003	0.933	0.890	0.975	2.12
Distance of aortic arch	0.495	0.159	3.112	0.002	1.640	1.208	2.260	1.52
Distance of total thoracic aorta	−0.299	0.103	−2.911	0.004	0.741	0.603	0.904	1.29
Tortuosity of ascending aorta	−5.453	1.646	−3.312	0.001	0.004	0.000	0.094	1.17
Tortuosity of aortic arch	−3.253	1.376	−2.364	0.018	0.039	0.002	0.529	1.28
Tortuosity of distal descending aorta	−5.056	1.563	−3.235	0.001	0.006	0.000	0.122	1.09
Type III arch (*n*, %)	−1.513	0.545	−2.775	0.006	0.220	0.073	0.626	2.3

## Data Availability

The data are available from the corresponding author upon reasonable request.

## References

[1] Thakkar D., Dake M.D. (2018). Management of Type B Aortic Dissections: Treatment of Acute Dissections and Acute Complications from Chronic Dissections. Tech. Vasc. Interv. Radiol..

[2] Riambau V., Böckler D., Brunkwall J., Cao P., Chiesa R., Coppi G., Czerny M., Fraedrich G., Haulon S., Jacobs M.J. (2017). Editor’s Choice—Management of Descending Thoracic Aorta Diseases: Clinical Practice Guidelines of the European Society for Vascular Surgery (ESVS). Eur. J. Vasc. Endovasc. Surg..

[3] Reutersberg B., Salvermoser M., Trenner M., Geisbüsch S., Zimmermann A., Eckstein H.H., Kuehnl A. (2019). Hospital Incidence and In-Hospital Mortality of Surgically and Interventionally Treated Aortic Dissections: Secondary Data Analysis of the Nationwide German Diagnosis-Related Group Statistics from 2006 to 2014. J. Am. Heart Assoc..

[4] Nomura Y., Nagao K., Hasegawa S., Kawashima M., Tsujimoto T., Izumi S., Matsumori M., Murakami H., Honda T., Mukohara N. (2019). Fatal Complications of New-Onset Complicated Type B Aortic Dissection After Endovascular Abdominal Aortic Aneurysm Repair: Report of 2 Cases and Literature Review. Vasc. Endovasc. Surg..

[5] Bossone E., Eagle K.A. (2021). Epidemiology and management of aortic disease: Aortic aneurysms and acute aortic syndromes. Nat. Rev. Cardiol..

[6] Heuts S., Adriaans B.P., Gerretsen S., Natour E., Vos R., Cheriex E.C., Crijns H.J.G.M., E Wildberger J., Maessen J.G., Schalla S. (2018). Aortic elongation part II: The risk of acute type A aortic dissection. Heart.

[7] Higashigaito K., Sailer A.M., van Kuijk S., Willemink M.J., Hahn J.D., Hastie T.J., Miller D.C., Fischbein M.P., Fleischmann D. (2021). Aortic growth and development of partial false lumen thrombosis are associated with late TBADverse events in type B aortic dissection. J. Thorac. Cardiovasc. Surg..

[8] Hiratzka L.F., Bakris G.L., Beckman J.A., Eagle K.A., Hermann L.K., Isselbacher E.M., Kazerooni E.A., Kouchoukos N.T., Lytle B.W., Milewicz D.M. (2010). 2010 ACCF/AHA/AATS/ACR/ASA/SCA/SCAI/SIR/STS/SVM guidelines for the diagnosis and management of patients with thoracic aortic disease. J. Am. Coll. Cardiol..

[9] Linda A.P., Thomas T.T., Eric M.I. (2007). Aortic diameter ≥ 5.5 cm is not a good predictor of type a aortic dissection: Observations from the International Registry of Acute Aortic Dissection (IRAD). Circulation.

[10] Qiu P., Liu J., Chen Y., Zha B., Ye K., Qin J., Hao P., Kang J., Zhang C., Zhu H. (2020). Changes in aortic arch geometry and the risk for Stanford B dissection. J. Thorac. Dis..

[11] Komutrattananont P., Mahakkanukrauh P., Das S. (2019). Morphology of the human aorta and age-related changes: Anatomical facts. Anat. Cell Biol..

[12] Redheuil A., Yu W.C., Mousseaux E., Harouni A.A., Kachenoura N., Wu C.O., Bluemke D., Lima J.A. (2011). Age-related changes in aortic arch geometry: Relationship with proximal aortic function and left ventricular mass and remodeling. J. Am. Coll. Cardiol..

[13] Adriaans B.P., Heuts S., Gerretsen S., Cheriex E.C., Vos R., Natour E., Maessen J.G., Nia P.S., Crijns H.J., Wildberger J.E. (2018). Aortic elongation part I: The normal aortic ageing process. Heart.

[14] Huang Y.-Q., Liang C.-H., He L., Tian J., Liang C.-S., Chen X., Ma Z.-L., Liu Z.-Y. (2016). Development and Validation of a RTBADiomics Nomogram for Preoperative Prediction of Lymph Node Metastasis in Colorectal Cancer. J. Clin. Oncol..

[15] Debray T.P., Collins G.S., Riley R.D., Snell K.I., Van Calster B., Reitsma J.B., Moons K.G. (2015). Transparent reporting of a multivariable prediction model for individual prognosis or diagnosis (TRIPOD): The TRIPOD statement. BMJ.

[16] Kramer A.A., Zimmerman J.E. (2007). Assessing the calibration of mortality benchmarks in critical care: The Hosmer–Lemeshow test revisited. Crit. Care Med..

[17] Howard D.P., Banerjee A., Fairhead J.F., Perkins J., Silver L.E., Rothwell P.M. (2013). Population-based study of incidence and outcome of acute aortic dissection and premorbid risk factor control: 10-year results from the Oxford Vascular Study. Circulation.

[18] De Martino A., Morganti R., Falcetta G., Scioti G., Milano A.D., Pucci A., Bortolotti U. (2019). Acute aortic dissection and pregnancy: Review and meta-analysis of incidence, presentation, and pathologic substrates. J. Card. Surg..

[19] Akutsu K. (2019). Etiology of aortic dissection. Gen. Thorac. Cardiovasc. Surg..

[20] Adriaans B.P., Wildberger J.E., Westenberg J.J., Lamb H.J., Schalla S. (2019). Predictive imaging for thoracic aortic dissection and rupture: Moving beyond diameters. Eur. Radiol..

[21] Poullis M.P., Warwick R., Oo A., Poole R.J. (2008). Ascending aortic curvature as an independent risk factor for type A dissection, and ascending aortic aneurysm formation: A mathematical model. Eur. J. Cardiothorac. Surg..

[22] Elefteriades J.A. (2002). Natural history of thoracic aortic aneurysms: Indications for surgery, and surgical versus nonsurgical risks. Ann. Thorac. Surg..

[23] Ohyama Y., Redheuil A., Kachenoura N., Ambale Venkatesh B., Lima J.A. (2018). Imaging Insights on the Aorta in Aging. Circ. Cardiovasc. Imaging.

[24] Hiratzka L.F., Bakris G.L., Beckman J.A., Bersin R.M., Carr V.F., Casey Jr D.E., Eagle K.A., Hermann L.K., Isselbacher E.M., Writing Group Members (2010). ACCF/AHA/AATS/ACR/ASA/SCA/SCAI/SIR/STS/SVM guidelines for the diagnosis and management of patients with Thoracic Aortic Disease: A report of the American College of Cardiology Foundation/American Heart Association Task Force on Practice Guidelines, American Association for Thoracic Surgery, American College of Radiology, American Stroke Association, Society of Cardiovascular Anesthesiologists, Society for Cardiovascular Angiography and Interventions, Society of Interventional Radiology, Society of Thoracic Surgeons, and Society for Vascular Medicine. Circulation.

[25] Waterford S.D., Di Eusanio M., Ehrlich M.P., Reece T.B., Desai N.D., Sundt T.M., Myrmel T., Gleason T.G., Forteza A., de Vincentiis C. (2017). Postoperative myocardial infarction in acute type A aortic dissection: A report from the International Registry of Acute Aortic Dissection. J. Thorac. Cardiovasc. Surg..

[26] Mehta R.H., Suzuki T., Hagan P.G., Bossone E., Gilon D., Llovet A., Maroto L.C., Cooper J.V., Smith D.E., Armstrong W.F. (2002). Predicting death in patients with acute type A aortic dissection. Circulation.

[27] Tolenaar J.L., Froehlich W., Jonker F.H., Upchurch R.G., Rampoldi V., Tsai T.T., Bossone E., Evangelista A., O’Gara P., Pape L. (2014). Predicting in-hospital mortality in acute type B aortic dissection: Evidence from International Registry of Acute Aortic Dissection. Circulation.

[28] Guo T., Fang Z., Yang G., Zhou Y., Ding N., Peng W., Gong X., He H., Pan X., Chai X. (2021). Machine learning models for predicting in-hospital mortality in acute aortic dissection patients. Front. Cardiovasc. Med..

